# Lineage switch from lymphoma to myeloid neoplasms: First case series from a single institution

**DOI:** 10.1515/med-2022-0521

**Published:** 2022-09-08

**Authors:** Wenjuan Yang, Shuangfeng Xie, Yiqing Li, Jieyu Wang, Jie Xiao, Kezhi Huang, Xiuju Wang, Yudan Wu, Liping Ma, Danian Nie

**Affiliations:** Department of Hematology, Guangdong Provincial Key Laboratory of Malignant Tumor Epigenetics and Gene Regulation, Guangdong-Hong Kong Joint Laboratory for RNA Medicine, Sun Yat-Sen Memorial Hospital, Sun Yat-Sen University, Guangzhou 510120, PR China; Department of Hematology, Sun Yat-Sen Memorial Hospital, Sun Yat-Sen University, Guangzhou 510120, PR China

**Keywords:** lineage switch, lymphoma, myeloid neoplasms

## Abstract

Lymphoma relapse is very common in clinical work, but lineage switch at relapse is rare. Although some cases have reported acute lymphocytic leukemia (ALL) switch to acute myeloid leukemia (AML) or myeloid sarcoma upon relapse, phenotype switch seldom occurs in other types of lymphoma. Here we report six cases with lineage switch from lymphoma to myeloid neoplasms. In our cohort, three cases were mantle cell lymphoma (MCL), and the other three cases were T-cell lymphoblastic lymphoma (T-LBL), B-cell lymphoblastic lymphoma (B-LBL), and diffuse large B-cell lymphoma (DLBCL) at the initial diagnosis. When linage switch occurred, most cases were AML M5 phenotypes, and only one case was myelodysplastic syndrome (MDS) phenotype. 11q23/mixed-lineage leukemia (MLL) rearrangement was negative in all cases. Although intensive therapy and stem cell transplantation have been applied in most cases, the poor outcome cannot be reversed. Therefore, we found that lineage switch could occur not only from ALL to AML or *vice versa*, but also from MCL or DLBCL to AML. Moreover, the incidence of MLL rearrangement in lineage switch is lower in adult hematologic malignancies as compared with pediatric patients.

## 1 Introduction

Leukemia relapse/refractory presents a tough challenge for hematologists [1]. Leukemia stem cells (LSCs), which are derived from hematopoietic stem cells (HSCs), multipotent progenitors, committed progenitor cells, or even differentiated cells that undergo gene mutation, play a vital role in leukemia initiation and progression [2]. LSCs also exhibit lineage plasticity and reversibility due to lineage-specific transcription factors’ mutation, changes in cytokine profiles from microenvironment, and therapy [3,4,5]. Upon relapse, a phenomenon termed “lineage switch” is observed in a subset of leukemia patients [6].

Lineage switch rarely occurred in leukemia relapse/refractory patients, but it can result in high mortality rate. A recent study showed that the incidence of lineage switch, most with 11q23/mixed-lineage leukemia (MLL) rearrangement, was 0.6% in patients with childhood acute leukemia [6]. In addition, there are several casereports on lineage switch in adultleukemia. Lineage switch primarily occurs from acute lymphocytic leukemia (ALL) to acute myeloid leukemia (AML) [7,8,9,10,11], but some cases switched from AML to ALL [12], as well as from T-cell acute leukemia to B-cell acute leukemia [13], or repeatedly switched in T-cell lymphoblastic lymphoma (T-LBL)/ALL and AML [14]. Although studies have found that cell fate regulators, cytokine profiles, and treatment modalities can play a role in lineage switch, the mechanism of the process remains unclear [3,4]. Therefore, describing diverse leukemia cases with lineage switch in detail will deepen the understanding of rare lymphoma recurrence/refractory cases and help to develop new treatment strategies in the future.

In the present study, we reported six cases of lineage switch that occurred in adult LBL and B-cell lymphoma (mantle cell lymphoma [MCL] and diffuse large B-cell lymphoma [DLBCL]) patients. This is the largest case series studies in adults from a single institute. Unlike previous reported pediatric cases, 11q23/MLL rearrangement was negative in our cases. When lineage switch occurred, most of our cases were AML-M5 phenotypes.

## 2 Case presentation

### 2.1 Case 1

A 54-year-old male presented with abdominal distension and visited the local hospital in Jan 2013. Colon neoplasm was discovered by ultrasound. After colonoscopy and biopsy, immunohistochemistry (IHC) results showed the tumor cells expressed CD20, CD79a, BCL-2, CD5, and CyclinD1. Ki67 was positive in 20% of neoplastic cells. The patient was diagnosed with MCL and received 8 cycles of R-CHOP regimen. But the patient relapsed after 2 years of follow-up. Positron emission tomography/computer tomography (PET/CT) showed a retroperitoneal mass, and the high standardized uptake value (SUV) on bone marrow (BM). MCL relapse was confirmed in mass biopsy by IHC staining for CD20, CD79a, BCL2, CD5, and CyclinD1. BM morphology showed lymphoma invasion. The patient received 4 cycles of treatment (bortezomib, lenalidomide, and rituximab). Treatment response evaluation showed partial remission. After 2 months, blood test showed WBC 1.43 × 10^9^/L, HGB 66 g/L, PLT 171 × 10^9^/L. BM morphology showed 25% promonocytes, immunophenotype revealed diffuse infiltration of blast cells, expressing HLA-DR, CD11c, CD36, and CD64. Promonocytes expressed CD64 and CD56. 11q23/MLL rearrangement was negative. Cytogenetic analysis was normal. A diagnosis of AML (M5) was established and the patient started cladribine combine IA (idarubicin and cytarabine) for induction therapy. Post-treatment PET-CT scan showed multiple enlarged and hyper-metabolic lymph nodes throughout the whole body. There were no blast cells in BM morphology and immune-phenotype analysis, and the patient underwent allogeneic peripheral blood stem cell transplantation (allo-PBSCT). After 15 months, the patient presented with enlarged cervical lymph nodes. Due to progression of MCL (MCL) and pulmonary infection, the patient expired 34 months after his original diagnosis.

### 2.2 Case 2

A 32-year-old male presented with cough and shortness of breath. His symptoms occurred over 4 weeks beginning in March 2015. His chest CT showed a mediastinal mass and pleural effusion on the left chest. The mass biopsy by IHC staining results showed tumor cells expressing TDT, CD2, CD3, CD5, CD7, and BCL2, but negative for CD20, CD79a, CCND1, CD10, CD23, and BCL6. BM morphology and immunophenotype revealed 10% of blast cells with the same phenotypes. According to these results, the patient was diagnosed as T-LBL/ALL (IVA). He accepted induction therapy with BFM95 regimen and achieved complete remission (CR). Then, the patient received consolidation and maintenance therapy according to the protocol, along with prophylactic intrathecal lumbar injections for ten times. In August 2016, BM morphology and immunophenotype revealed 6% myeloid blast cells, expressing CD117, CD33, CD13, CD38, CD7, and partly expressing CD71, CD34, and HLA-DR, but negative for MPO, TDT, CD3, and CD79a. Molecular analysis showed TED2 and ASXL1 mutation. Chromosome karyotype and 11q23/MLL rearrangement were normal. The patient received sibling donor allo-PBSCT. After 4 years of follow-up, the patient relapsed in May 2020. BM morphology and immunophenotype revealed T-ALL/LBL relapse. Due to progression of disease and pulmonary infection, the patient expired 63 months after his original diagnosis.

### 2.3 Case 3

A 54-year-old female presented with chest pain for 1 week in September 2016. Her chest CT revealed mediastinal mass with swelling of multiple lymph nodes. Mass biopsy by IHC staining results showed tumor cells expressing TDT, CD19, BCL2, PAX5, but negative for CD3, CD5, CD20, CD10, BCL6, CD21, CD23, CyclinD1, and EBER. Proliferative index of Ki67 showed 70% positive. MYC rearrangement was negative. BM morphology, immunophenotype, and cytogenetic analysis were normal. A diagnosis of B-cell lymphoblastic lymphoma (B-LBL) (IIIA) was made. The patient started treatment with hyperCVAD/MA regimens for three cycles, as well as intrathecal lumbar injection for six times. In April 2017, BM morphology revealed 35% blast cells, immunophenotype showed blast cells expressed HLA-DR, CD33, CD11b, CD15, CD36, CD64, CD14, and CD13, but negative for CD34 and CD117. Molecular analysis showed CBL mutation, there were no TET2, SRSF2, and ASXL1 mutations. Chromosome karyotype and 11q23/MLL rearrangement were normal. A diagnosis of AML (M5) was established and the patient started IA regimen for induction therapy. The patient obtained CR and received D-HAG (decitabine, homoharringtonine, cytarabine, and G-CSF) regimen for consolidation therapy. After that, the patient underwent allo-PBSCT. At present, 58 months from her original diagnosis, the patient has achieved continuous CR status.

### 2.4 Case 4

A 62-year-old female presented with hematuria for 3 months in July 2018. Ultrasound scan showed calculus of kidney and enlarged inguinal lymph nodes. The lymph node biopsy by IHC staining results showed tumor cells were positive for CD20, CD79a, CyclinD1, and SOX11, but negative for CD3, CD10, and BCL6. Proliferative index of Ki67 showed 25% positive. BM morphology and immunophenotype analysis revealed that 80% myeloid blast cells expressing CD117, CD33, CD56, CD13, and CD64, but negative for CD34, HLA-DR, CD19, CD16, CD14, and CD20. BM histology revealed diffuse infiltration of blast cells, expressing MPO and CD117, but negative for CD20, SOX11, CyclinD1, CD10, TDT, and CD34. Next generation sequencing (NGS) showed CEBPA, FLT3-ITDlow, TET2, and NPM1 mutations. Chromosome karyotype was normal and 11q23/MLL rearrangement was negative. PET-CT scan showed multiple cervical, axillary and inguinal lymph nodes up to 3 cm, the maximal SUV (SUVmax) was 2.7 with splenomegaly. The diagnoses were: (1) AML (M5b, with CEBPA, FLT3-ITDlow, TET2, and NPM1 mutation; low risk group) and (2) MCL (MIPI score was 3; low risk group). The patient started induction therapy with IA regimen. After induction therapy, the minimum residual disease (MRD) of BM showed there were no myeloid blast cells, but CD19 and CD20 positive B lymphocytes accounted for 10%, expressing FMC7, CD5, CD22, and CD79a, but negative for CD23. We then used rituximab and high-dose cytarabine for consolidation therapy for four cycles. After consolidation therapy, MRD was negative, and no enlarged lymph nodes were found on PET-CT. Then, the patient underwent autologous peripheral blood stem cell transplantation (auto-PBSCT). At present, 36 months from her original diagnosis, the patient has achieved continuous CR status.

### 2.5 Case 5

A 52-year-old female presented with abdominal mass in December 2015. Mass biopsy by IHC staining results showed tumor cells expressing LCA, CD20, CD79a, Mum1, and BCL6, but negative for CD10, CD3, CD5, P53, and BCL2. Proliferative index of Ki67 showed 90% positive. MYC rearrangement was negative. A diagnosis of diffuse large B cell lymphoma (non-GCB, IA, IPI 0 score) was made, and the patient received 6 cycles of R-EPOCH treatment from January to July 2016. The efficacy was evaluated by PET-CT scan after 2 and 6 cycles of treatment and showed CR at both times. In December 2017, the patient presented with fever, and peripheral blood count showed WBC 118 × 10^9^/L, HGB 74 g/L, PLT 44 × 10^9^/L. The morphological examination of BM aspirate showed mixture of myeloblasts (70%) plus monoblasts and promonocytes (24%). Immunophenotype analysis showed 87.6% myeloid blast cells, expressing CD117, CD33, CD38, and partly expressing CD64 and CD56, but negative for TDT, MPO, CD13, CD34, HLA-DR, CD19, CD14, and CD20. Chromosome karyotype was normal and 11q23/MLL rearrangement was negative. NGS revealed FLT3-ITD, TET2, and NPM1 mutations. The patient was diagnosed with AML (M4, with FLT3-ITD, TET2, and NPM1 mutation; intermediate-risk group), and used IA regimen for induction therapy. After one cycle of therapy, BM aspirate showed no remission (NR), so the patient was given sorafenib combined CLAG regimen (cladribine, cytarabine, and G-CSF) for salvage therapy. Then, the patient achieved CR and MRD test was negative. The patient had no desire for HSC transplantation and there was no available donor. Therefore, we continued to administer HD Ara-C for consolidation therapy and the total 6 cycles of treatments were finished in November 2018. 27 months later, the patient presented with fever and enlarged inguinal lymph nodes. PET-CT scan showed hypermetabolism of inguinal lymph nodes, the maximum volume was 1.5 cm × 2 cm. Lymph nodes biopsy by IHC staining results showed inflammation, but there were no tumor cells. BM morphology and immunophenotype revealed AML relapse, chromosome karyotype showed 46, XX, t(1:8)(q31:q22) [10]. NGS revealed CBL, TET2, and NPM1 mutations. The patient then started CLAG combined mitoxantrone treatment and achieved CR again. At present, 6 months from her second relapse, the patient has achieved continuous CR status.

### 2.6 Case 6

A 50-year-old male presented with abdominal pain for 6 months in May 2015. Gastroscopy and biopsy were conducted, and IHC staining results showed the tumor cells were positive for CD20, CD79a, CyclinD1, and BCL2, but negative for CD3, CD10, and HP. Proliferative index of Ki67 showed 50% positive. A diagnosis of MCL was made and the patient started treatment with RCHOP regimen for six cycles. After chemotherapy, the gastroscopy and pathological examination still showed residual MCL tumor cells. The efficacy was evaluated as partial response, so the patient underwent 8 doses of rituximab for maintenance therapy and involved-site radiotherapy for 40GY. Regular follow-up showed no disease progression. After 2 years of observation, the patient presented with abdominal distension and CT scan showed tumor progression. Two cycles of GemOx (gemcitabine and oxaliplatin) regimen were used as salvage therapy. Three months later, the blood test showed WBC 124 × 10^9^/L, HGB 66 g/L, PLT 81 × 10^9^/L. BM morphology and immune-phenotype revealed AML (M5), NGS showed FLT3-ITD high mutation. Chromosome karyotype showed 46 XY, t(2:8) (p13:p21) del(9)(q22) [20]. 11q23/MLL rearrangement was negative. The patient received IA for induction therapy, but efficacy evaluation was no response. Then, sorafenib and venetoclax were used for salvage therapy, but the rapid agranulocytosis and infection resulted in death 20 days after chemotherapy was begun.

This study was reviewed and approved by the ethics review boards of Sun Yat-Sen Memorial Hospital, the informed consent has been obtained from all individuals included in this study.

## 3 Discussion

Cases of lineage conversions from lymphoma to myelodysplastic syndrome (MDS) or AML are extremely rare. In the literature, most of those cases were switches from ALL to AML [7,8,9,10,11], switches from AML to ALL [12] was also reported. But in our cohort, three cases were MCL lymphoma, and the other three cases were T-LBL, B-LBL, and DLBCL at initial diagnosis (Table 1). When lineage switch occurred, four cases were AML-M5 phenotypes (Table 1). Analysis of these special subsets not only let us better understand the mechanism of tumor progression, but also helped clinicians to explore more effective therapy for patients.

**Table 1:** Characteristics of our cases and reported cases

	Age/Sex	1st diagnosis	2nd diagnosis	Interval of two disease	Disease progression	Outcome	Overall survival
**Our cases**							
Case 1	54/M	MCL	AML M5	34 mo	MCL	Died	52 mo
Case 2	32/M	T-LBL	MDS EB1	17 mo	T-LBL/ALL	Died	63 mo
Case 3	47/F	B-LBL	AML M5	8 mo		Alive	58 mo
Case 4	62/F	MCL	AML M5	7 dy		Alive	36 mo
Case 5	52/F	DLBCL	AML M4	24 mo	AML M4	Alive	67 mo
Case 6	55/M	MCL	AML M5	24 mo	AML M4	Died	56 mo
**Reported cases [References]**							
[7]	25/F	Pro-B ALL	AML M5	22 mo	AML M5b	Died	24 mo
[8]	60/F	Pre-B ALL	AML M5	42 dy	AML M5	Died	44 dy
[9]	30/M	B-LBL	AML M5	1 mo	B-LBL&AML M5	Died	2 mo
[10]	20/M	T-ALL	AML	21 mo	AML M5	Died	30 mo
[11]	40/F	Pro-B	AML	40 mo	AML	Died	42 mo
[12]	46/M	AML M5	Pre-B ALL	10 mo	B-ALL	Died	20 mo
[13]	31/M	T-ALL	B-ALL	26 mo	AML M5	Alive	64 mo
[5]	77/M	B-ALL	Myeloid sarcoma	8 mo	Myeloid sarcoma	No data	No data
[14]	32/M	T-LBL	AML	1 mo	T-LBL	Died	9 mo

Abbreviation: M: male, F: female, MCL: mantle cell lymphoma, AML: acute myeloid leukemia, T-LBL: T cell lymphoblastic lymphoma, B-LBL: B-cell lymphoblastic lymphoma, DLBCL: diffuse large B-cell lymphoma, ALL: acute lymphocytic leukemia, mo: months, dy: days.

Some hypotheses may explain the mechanism of lineage switch. First, chemotherapy can screen out few drug-resistant clones. After several cycles of treatment, the small size of clone gradually converted into predominant clones and then phenotype switch happened [15]. Second, gene reprogramming happened at the level of neoplastic pluripotent stem cells, or the existence of bipotential lympho-myeloid progenitor cells. Third, lymphoid phenotype developed into myeloid phenotype through trans-differentiation or indirect dedifferentiation and differentiation. One study showed that cells from earliest thymic progenitors (ETPs) possess the ability to differentiate into T and natural killer lymphoid lineages, and few subsets of ETPs have the potential to differentiate into B lymphoid lineages [16]. Another study focused on the fact that most ETPs possess T and myeloid potential, so the author refuted the idea of an obligatory initial split in hematopoiesis between lymphoid lineages and myelo-erythroid lineages. Finally, ectopic expression or loss of lineage-specific transcription factor such as Pu.1, C/ebp-a, Gata1, Pax5, and Ikaros are often related with a changed network of transcription factors that control cell fate [3,4]. BM cytokine milieu and metabolic parameters of LSCs also profoundly affect their lineage commitment [17,2].

While LSCs are critical in the maintenance of leukemia, the existence of lymphoma stem cells remains uncertain. In B cell lymphoma, cloned translocation involved with immunoglobulin loci related to the pathogenesis of B-cell malignancies, but there is no defined role in stem cell characteristics [18,19]. In our cohort, the initial phenotype of three cases were MCL and 1 case was DLBCL, and none of the patients were positive for MYC rearrangement. Some researchers considered that when lymphoma-initiating cells acquire stem-cell features though secondary reprogramming mutations to become the lymphoma-originating cells, they were fully capable of generating and maintaining lymphomas [20]. Some cells such as centroblasts and memory B cells have certain stem-cell-like features [21,22], as well as lymphocytes with MYC rearrangements may directly function as the lymphoma-originating cells [20]. So, we considered those theories as applying to our MCL cases. Moreover, it is worth to study why monocytic phenotypes are more common in lineage switch.

Some studies suggest that MLL rearrangement plays a certain role in lineage switch. In the literature, lineage switch with MLL positive cases were more common in pediatric acute leukemia [1]. MLL rearranged leukemia seemed to possess lineage plasticity, and they were able to differentiate myeloid or lymphoid lineage through different cytokines culture conditions [23,24]. Microenvironmental cues can cooperate with MLL fusion proteins to determine lineage outcomes in leukemia of CD34 positive multipotent progenitors as well as CD19 or CD33 positive progenitors that have partially differentiated [25]. However, in our cohort, no patient showed positive for MLL rearrangement. From the data of nine reported cases, there was only one patient who showed MLL rearrangement (Table 2, ref. [5]). Therefore, we could conclude that the incidence of MLL rearrangements in lineage switch is lower in adult hematologic malignancies.

**Table 2:** MICM information and treatment regimen of our cases and reported cases

	Morphology or immunophenotype	Cytogenetics	Molecular biology (mutation)	Chemotherapy regimen	Stem cell transplantation
**Our cases**					
1 Diagnosis	MCL	46,XY [20]	No data	RCHOP, RFC, R2 + bortezomib, IR + bortezomib	
1 Switch	AML M5	46,XY [20]	NPM1, TET2, ASXL1	CLAG, IA	Unrelated donor PBSCT
2 Diagnosis	T-LBL	46,XY [20]	Negative	BFM95	
2 Switch	MDS	46,XY [20]	TET2, ASXL1 (SNP cites)		Sibling donor PBSCT
3 Diagnosis	B-LBL	46,XX [20]	Negative	Hyper CVAD A/B	
3 Switch	AML M5	46,XX [20]	CBL	IA, D-HAG	Sibling donor PBSCT
4 Diagnosis	MCL	46,XX [20]	Negative	IA, IA + VP-16	
4 Switch	AML M5	46,XX [7]	CEBPA, NPM1, TET2, FLT3-ITDlow	R + HDAra-C	Auto-PBSCT
5 Diagnosis	DLBCL	46,XX [20]	Negative	REP OCH + RT	
5 Switch	AML M4	t(1:8)(q31;q22) [17]/46,XX [3]	FLT3-ITD, TET2, NPM1, CEBPA	IA, Sorafenib + CLAG, HDArc-a	
6 Diagnosis	MCL	46,XY [20]	Negative	RCHOP, RT, GEMOX	
6 Switch	AML M5	46 XY? t(2:8)(p13:p21) del(9)(q22) [20]	FLT3-ITDhigh	IA, Sorafenib, Venetoclax	
**Reported case [Reference]**					
[7] Diagnosis	Pro-B ALL	46,XY [10]	TAF15 – ZNF384	(GIMEMA) LAL 2000	
[7] Switch	AML M5b	47,XX, t(12;17)(p13;q11) mar [6]/46,XX [6]	TAF15 – ZNF384	FLAG	
[8] Diagnosis	Pre-B ALL	No data	Negative	VCDLP	
[8] Switch	AML M5	No data		FLAG	
[9] Diagnosis	B-LBL	46,XX	IGH rearrangement, MRP1, and BCRP1 overexpress	GMALL-2003 protocol	
[9] Switch	AML M5	No data	EVI-1	IDA-FLAG	
[10] Diagnosis	T-ALL	52, XY, +? X, + 8, + 10, + 11, + 13, + 19 [11]/46, XY [9]	Negative	HOVON 70	
[10] Switch	AML	No data	Negative	HD Ara-c + VP16	Auto-PBSCT
[11] Diagnosis	Pro-B ALL	t(2;16) (p11;p11) [10]	SMC3	GIMEMA 0904 protocol	
[11] Switch	AML	t(2;16) (p11;p11), + 12[13] no data	SMC3, ETV6, JARID2, KLF4, PIK3C2A, PTPRG	Clofarabine and cytarabine	
[12] Diagnosis	AML(M4)	Normal	Negative	IA	Autologous bone marrow transplantation
[12] Switch	Pre-B ALL	Normal	Monoclonal pattern of IgH and TCR	VCDLP, MTX,6-MP	
[13] Diagnosis	T-ALL	46,XX [20]	No data	CTX + VDLD	
[13] Switch	B-ALL	No data	BCR/ABL P210 positive	No data	
[5] Diagnosis	B-ALL	46,XY,t(4;11)(q21;q23)	No data	Blinatumomab study (NCT02143414)	
[5] Switch	Myeloid sarcoma	No data	No data	No data	
[14] Diagnosis	T-LBL	46,XX	ALK, MAP3K14 mutation	AALL0434 protocol	
[14] Switch	AML	46,XX	ALK, MAP3K14 mutation	Cytarabine, mitoxantrone and etoposide, HDAra-c	Sibling ASCT

Abbreviation: PBSCT: peripheral blood stem cell transplantation, Auto-PBSCT: Autologous peripheral blood stem cell transplantation, ?: uncertain.

In three cases from our cohort, NGS revealed the most common mutations of AML such as FLT3-ITD, TET2, ASXL1, NPM1, C/EBPα, or CBL (Table 2). In our cases, one case was C/EBPα mutation and two cases showed CBL gene mutation. C/EBPα is one of the master transcription factors to regulate myeloid cell fate, and promotion of differentiation of progenitor cells and aberrant expression confer a myeloid cell fate to T cells, B cells, or fibroblasts [26,27]. At present, CBL gene mutation does not have a prognostic significance gene in adult AML case according to NCCN guidelines. FLT3-ITD, TET2, ASXL1, and NPM1 are well recognized in hematologic malignancies.

In terms of treatment, due to the high mortality of this disease, most of the cases in the literature ended in death. In our cohort, although the treatment was adjusted according to phenotype conversion, and allo- or auto-PBSCT was conducted according to the patient’s condition, but only three patients have survived. Thus, we need to make individualized treatment regimens in clinical practice. The reason for this is that we found that no one therapy regimen is more effective than another from our cohort. In the era of immunotherapy, it still needs to be explored whether antibody-based or cell-based therapy is a good choice for those patients.

## 4 Conclusion

From our case studies, we conclude that lineage switch is rare but leads to high-mortality incidence in hematology malignance. Lineage switch can occur not only from ALL to AML or *vice versa*, but also from MCL or DLBCL to AML. The incidence of MLL rearrangements in lineage switch is lower in adult hematologic malignancies compared with pediatric leukemia. Meanwhile, poor outcomes often cannot be reversed, although intensive therapy and stem cell transplantation have been used in some cases. Due to low incidence, the characteristics and gene expression signature of those cases varied greatly. As a result, we need to accumulate more cases to describe biological and clinical characteristics of this type of disease, as well as to explore more treatment options in order to improve clinical outcomes.
